# Switchable 3D Photonic Crystals Based on the Insulator-to-Metal Transition in VO_2_

**DOI:** 10.1021/acsami.4c13789

**Published:** 2024-12-02

**Authors:** Jun Peng, Julia Brandt, Maurice Pfeiffer, Laura G. Maragno, Tobias Krekeler, Nithin T. James, Julius Henf, Christian Heyn, Martin Ritter, Manfred Eich, Alexander Yu. Petrov, Kaline P. Furlan, Robert H. Blick, Robert Zierold

**Affiliations:** † Center for Hybrid Nanostructures, Universität Hamburg, Luruper Chaussee 149, 22607 Hamburg, Germany; ‡ Institute of Optical and Electronic Materials, Hamburg University of Technology, 21073 Hamburg, Germany; § Integrated Ceramic-Based Materials Systems Group, Hamburg University of Technology, 21073 Hamburg, Germany; ∥ Betriebseinheit Elektronenmikroskopie, Hamburg University of Technology, 21073 Hamburg, Germany; ⊥ Institute of Functional Materials for Sustainability, Helmholtz-Zentrum Hereon, 21502 Geesthacht, Germany; # Deutsches Elektronen-Synchrotron (DESY), Notkestrasse 85, 22607 Hamburg, Germany

**Keywords:** switchable photonics, vanadium dioxide, insulator-to-metal transition, atomic layer deposition, inverse opal

## Abstract

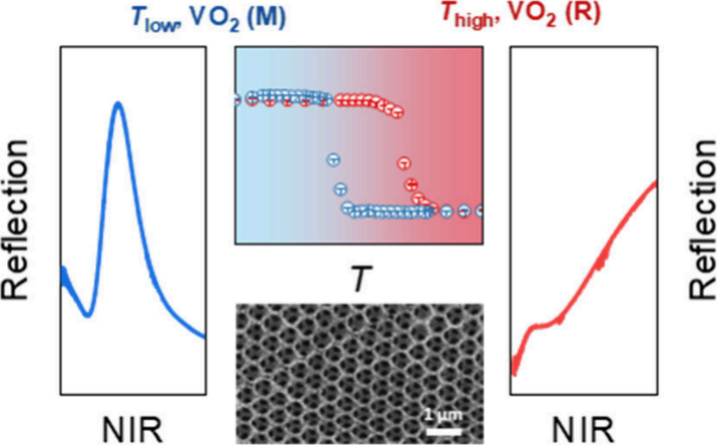

Photonic crystals (PhCs) are optical structures characterized by the spatial modulation of the dielectric function, which results in the formation of a photonic band gap (PBG) in the frequency spectrum. This PBG blocks the propagation of light, enabling filtering, confinement, and manipulation of light. Most of the research in this field has concentrated on static PhCs, which have fixed structural and material parameters, leading to a constant PBG. However, the growing demand for adaptive photonic devices has led to an increased interest in switchable PhCs, where the PBG can be reversibly activated or shifted. Vanadium dioxide (VO_2_) is particularly notable for its near-room-temperature insulator-to-metal transition (IMT), which is accompanied by significant changes in its optical properties. Here, we demonstrate a fabrication strategy for switchable three-dimensional (3D) PhCs, involving sacrificial templates and a VO_2_ atomic layer deposition (ALD) process in combination with an accurately controlled annealing procedure. The resulting VO_2_ inverse opal (IO) PhC achieves substantial control over PBG in the near-infrared (NIR) region. Specifically, the synthesized VO_2_ IO PhC exhibits PBGs near 1.49 and 1.03 μm in the dielectric and metallic states of the VO_2_ material, respectively, which can be reversibly switched by adjusting the external temperature. Furthermore, a temperature-dependent switch from a narrow-band NIR reflector to a broad-band absorber is revealed. This work highlights the potential of integrating VO_2_ into 3D templates in the development of switchable photonics with complex 3D structures, offering a promising avenue for the advancement of photonic devices with adaptable functionalities.

## Introduction

Photonics, the science and technology of generating, controlling, and detecting photons, have emerged as a cornerstone in modern engineering and scientific research. The manipulation of light has led to transformative advancements across various fields including telecommunications, sensing, imaging, and computing. Central to many of these advancements are PhCs—periodic nanostructures that possess unique optical properties due to their ability to control light propagation through PBGs and resonant modes,^1,2^ which are essential to various cutting-edge applications including metasurfaces, photonic integrated circuits, and photonic diagnosis.^3−5^ However, conventional static PhCs are limited by their lack of *in operando* tunability. This limitation restricts their adaptability and versatility, particularly in dynamic environments where the optical properties need to be adjusted in real-time in response to external stimuli.^6,7^ To address this challenge, researchers have focused on developing adjustable photonics, where the optical properties, such as the photonic band structure, resonance frequencies, and light propagation can be dynamically altered,^8,9^ enabling new possibilities for designing adaptive optical devices with enhanced functionality and performance.

VO_2_ stands out as a promising material for switchable photonics because of its near-room-temperature IMT, which is accompanied by significant changes in optical and electronic properties.^10−16^ In recent years, many studies have explored the use of VO_2_ to construct periodic structures or to integrate VO_2_ with such structures for various applications.^17−20^ For example, sol–gel infiltrated VO_2_ integrated into core/shell 2D PhC has been used to adjust the color for thermochromic smart window applications.^21^ Hybrids of crossed gold nanoantenna arrays and VO_2_ thin films synthesized by chemical vapor deposition have achieved reversible switching for nanoscale optical memory functionalities.^22^ Alternatively, sputtered VO_2_ thin films integrated with metal metasurfaces have been utilized as optical switches, optical limiters with adjustable thresholds, and nonlinear optical isolators.^23^ Despite these advancements, most VO_2_ integrations have been limited to 1D or 2D structures, with few attempts at 3D structures due to material synthesis challenges.^24−26^

As an advanced surface-limited coating technique, ALD offers precise conformality on 3D complex structures. Previous studies have demonstrated the deposition of VO_2_ thin films on a variety of planar substrates,^27−33^ indicating the potential for integrating VO_2_ into 3D photonic structures such as 3D PhCs. By exploiting the change in the optical parameters induced by the VO_2_’s IMT, the optical properties of integrated photonics can be dynamically modulated. In this work, we first demonstrate VO_2_ thin film synthesis via a combination of ALD and postdeposition annealing. The temperature-dependent IMT in the annealed thin film, transitioning from a low-temperature dielectric monoclinic phase VO_2_ (M) to a high-temperature rutile metallic phase VO_2_ (R), is investigated from electrical, structural, and optical perspectives. Subsequently, we successfully fabricated a VO_2_ IO PhC using a process adapted from the VO_2_ thin film deposition process to a polystyrene (PS) opal template. The fabricated VO_2_ IO PhC demonstrates PBGs at approximately 1.49 and 1.03 μm in the dielectric and metallic states of the VO_2_ material, respectively. Significantly, these PBGs can be reversibly switched by altering the external temperature. Additionally, a switch from a narrow-band NIR reflector to a broad-band absorber is observed in the dielectric and metallic state, respectively. The successful realization of VO_2_ IO PhC demonstrates the feasibility of integrating ALD-based VO_2_ with complex 3D structures, which opens up new opportunities for switchable photonics in adaptive and versatile optical devices.

## Results and Discussion

### VO_2_ Thin Film via ALD

**Figure 1:**
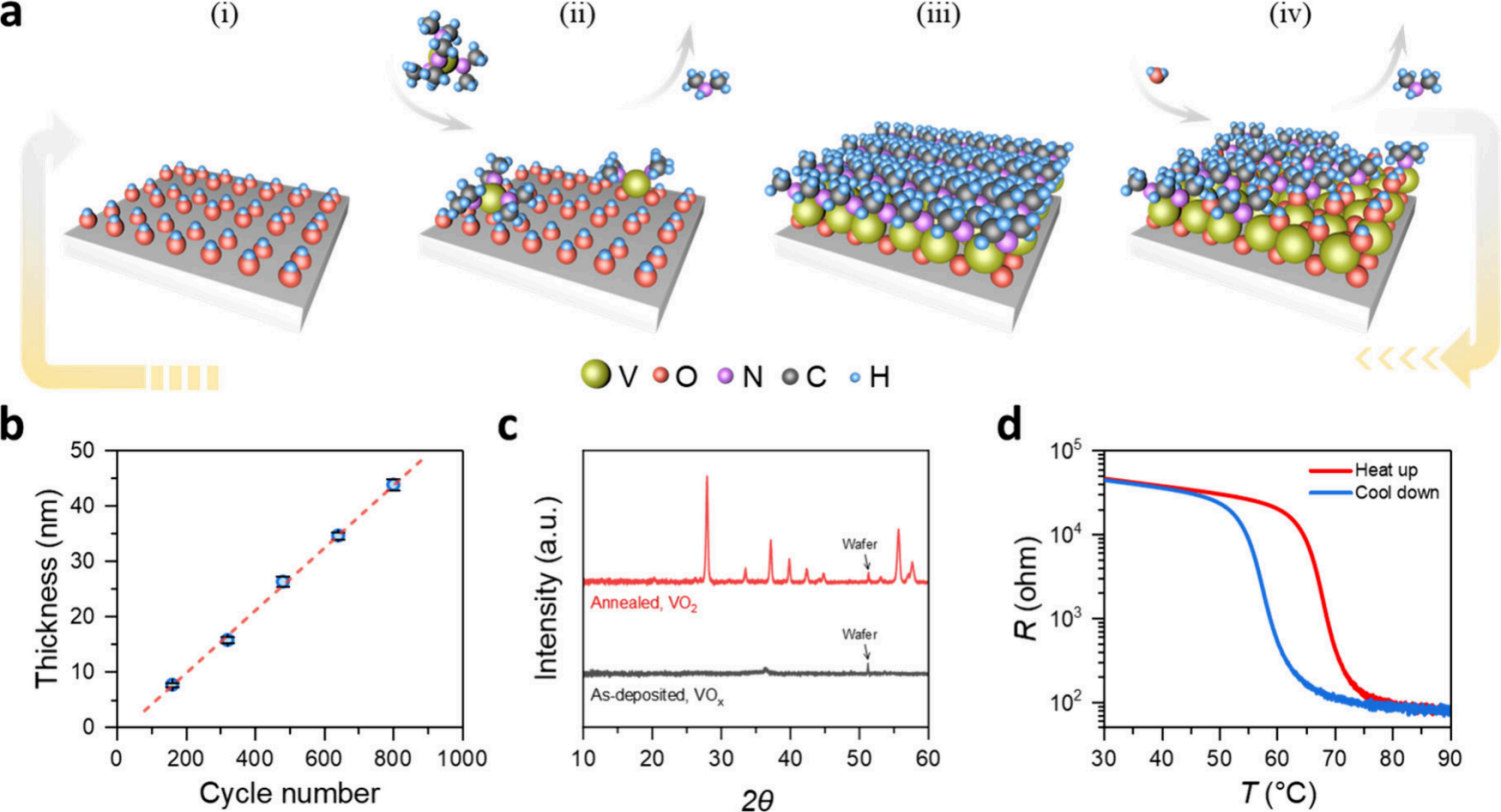
ALD deposition of VO_2_ thin film. (a) Mechanism of VO_x_ film deposition via ALD. This diagram illustrates the ligand-exchange process during the ALD cycles, showing how the surface becomes terminated with (i) −OH groups and (iii) −NMe_2_ groups after (iv) the H_2_O and (ii) the TDMAV pulses, respectively. (b) Thickness of the deposited films as a function of the number of ALD cycles, demonstrating a constant GPC of 0.56 Å/cycle. (c) GIXRD spectra for the as-deposited VO_x_ film and annealed VO_2_ film. In the annealed VO_2_ film, the peak near 2θ = 51° belongs to the substrate, while other peaks belong to VO_2_. A more detailed labeling is shown in Figure S1. (d) Temperature-dependent resistance measurement of the annealed VO_2_ film, revealing the IMT with over 3 orders of magnitude change in resistance.

The screening of suitable ALD precursors to later produce the 3D IO PhCs should meet two key conditions: (1) the precursor should not undergo chemical reactions with PS that would alter the polymer structure or degrade the template, and (2) ALD deposition should occur below the stable temperature for ALD processing of PS, which is approximately 100 °C. Attracted by the interesting IMT property, the ALD community has already reported several precursors for the successful synthesis of VO_x_ films, including VOCl_3_, VCl_4_, VO(OC_3_H_7_)_3_, tetrakis(ethylmethylamino)vanadium (V(NEtMe)_4_, TEMAV), tetrakis(dimethylamino)vanadium (V(NMe_2_)_4_, TDMAV).^26,34,35^ Among them, TEMAV has been the subject of most detailed studies.^27−30^ However, in the reported literature, only the combination of TDMAV and H_2_O has revealed a self-limited deposition of a dense VO_x_ film below 100 °C, which could then be converted into VO_2_ via a postdeposition annealing process.^32,33,36^ Therefore, we selected H_2_O and TDMAV as precursors for oxygen and vanadium sources, respectively. According to the ligand-exchange mechanism which is commonly suggested for dimethylamino metal precursors, e.g., Zr(NMe_2_)_4_, Zr(NEtMe)_4_, Hf(NMe_2_)_4_, Hf(NEtMe)_4_,^37,38^ the postulated deposition mechanism during the exposure ALD mode can be present in Figure 1a. During the first ALD half-cycle, the surface, saturated with hydroxyl groups (−OH) is exposed to and fully reacts with the TDMAV precursor, leaving −NMe_2_ groups at the surface. The byproduct HNMe_2_ and excessive TDMAV are pumped out in the following purging step. Then in the second half-cycle, the −NMe_2_ groups connected to the V atom react with the second precursor, H_2_O, forming a −OH group-terminated surface again after the H_2_O pulse and purge step. As the deposition cycles continue, the thickness of VO_x_ increases linearly with the number of cycles.

The deposition temperature is set at 95 °C, where a constant deposition rate, usually termed growth per cycle (GPC), of 0.56 Å/cycle is obtained (Figure 1b). In agreement to earlier reports,^32,33,36^ the as-deposited film is an amorphous VO_x_ film (Figure 1c and Figure S1a). After a postdeposition annealing process referring to the parameter’s ranges used for thermal treatments of VO_x_ deposited by TEMAV (see Material and Methods),^27,29^ the VO_x_ film is converted into a polycrystalline VO_2_ film in the monoclinic *P*2_1_/*c* space group (Figure 1c and Figure S1b). The annealing treatment not only adjusts the stoichiometry and crystallinity but also causes changes in the film morphology, i.e., in roughness and thickness. Specifically, for a 33.6 nm thick as-deposited film, its roughness increases from 0.57 to 1.63 nm after annealing (Figure S2); and for a thicker as-deposited film of 140 nm thickness, a shrinkage of about 2% of the height is observed after the same annealing process (Figure S3). This slight change in morphology can be attributed to the more regular and orderly arrangement of atoms in polycrystalline VO_2_ films compared with amorphous VO_x_ films after postdeposition annealing. The resistance change over temperature is one of the most important indicators of the VO_2_ IMT performance. The temperature-dependent resistance measurement (Figure 1d) exhibits an IMT with over 3 orders of magnitude, accompanied by a hysteresis width of 10.3 °C (Figure S4), which is comparable to VO_2_ synthesized by other synthetic methods, such as pulsed laser deposition^39^ and molecular beam epitaxy.^40,41^

### Structural Characterization of the VO_2_ Thin Film

**Figure 2:**
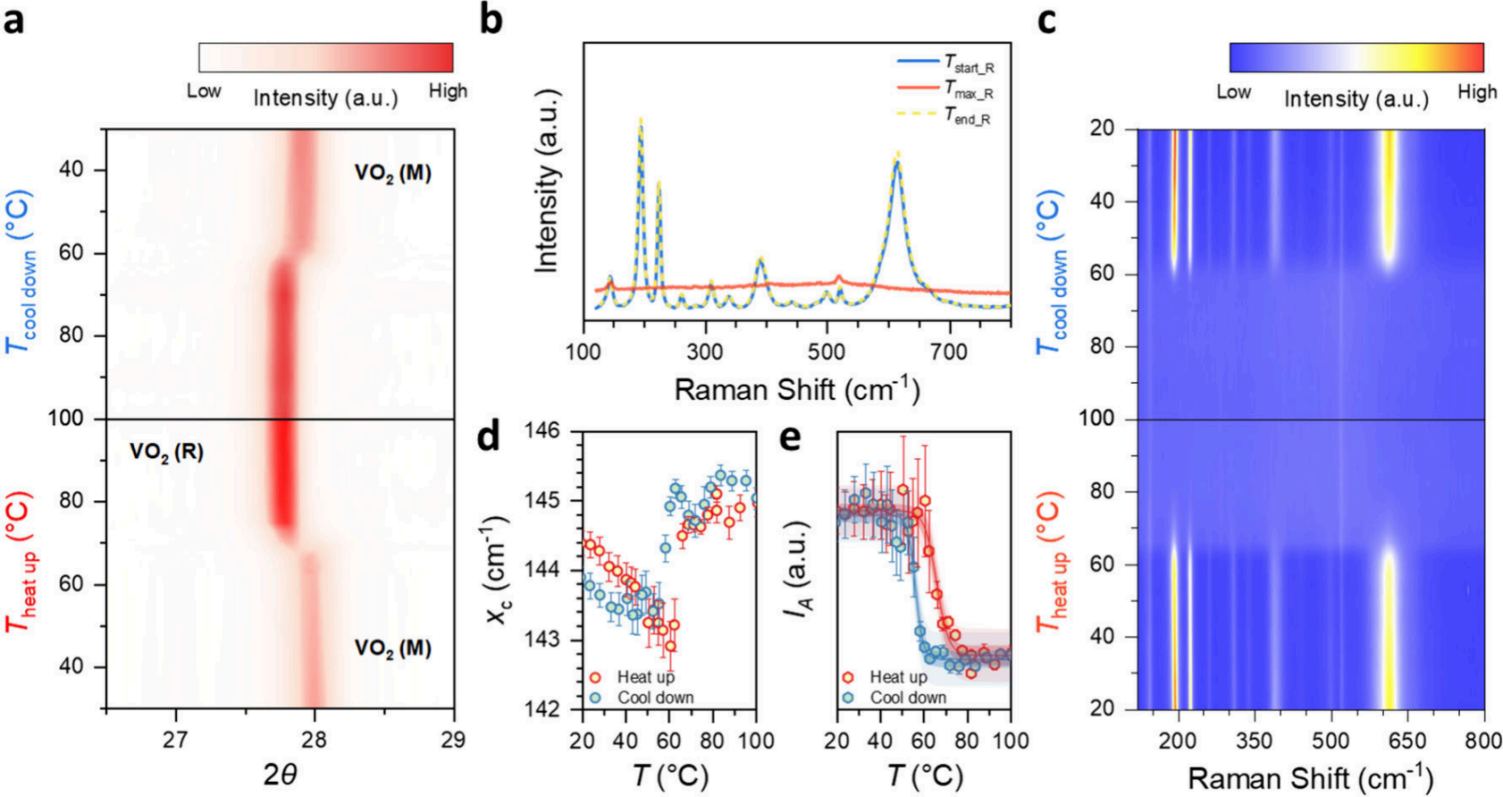
IMT characterization of VO_2_ thin film. (a) *In situ* temperature-dependent GIXRD measurement zoomed in near 2θ = 28° region. The peak shown belongs to the (011) plane of VO_2_ (M) at low temperature while the (110) plane of VO_2_ (R) at high temperature. (b) Raman spectra at low and high temperature. (c) Temperature-dependent Raman measurement. The peak at ∼520 cm^–1^, which remains almost unaltered, is the signal from the SiO_2_/Si substrate. Lorentz fitting analysis for (d) the peak position shift and (e) the peak area as peak intensity at ∼144.5 cm^–1^ as a function of temperature.

The negligible morphological change and impressive electrical IMT performance indicate the feasibility of ALD in constructing VO_2_-based 3D structures. Furthermore, the quality of the ALD-based VO_2_ thin films is verified by detailed grazing incidence X-ray diffraction (GIXRD) and Raman characterization during *in situ* heating and cooling processes. In the *in situ* temperature-dependent GIXRD measurement shown in Figure 2a, a small shift of approximately 0.15° is observed at around 68 and 60 °C during heating and cooling, respectively, for the most intense peak near 2θ = 28° representing the (011) plane of VO_2_ (M). The shifted peak at elevated temperatures represents the (110) plane of VO_2_ (R), indicating the expected reversible transition from the VO_2_ (M) phase to VO_2_ (R) phase.^28,42,43^ To ensure no other remanent phase changes occurred in the samples during the *in situ* experiments, the samples were also measured by GIXRD before and after the heating/cooling cycles covering a broader range of 2θ from 10° to 60°. The resulting diffractogram depicted in Figure S1b indicates a reversible IMT change without irreversible phase transformation within the film.

Additionally, Raman spectra of the annealed thin film at near room temperature, i.e., *T*_start_R_ = 19.4 °C and *T*_end_R_ = 19.1 °C in Figure 2b, exhibit distinct peaks at 144.5, 195.0, 224.8, 261.6, 285.7, 310.3, 338.9, 390.28, 440.2, 499.6, 613.8 cm^–1^, corresponding to the VO_2_ (M) phase, which agrees well with previous reports on crystalline VO_2_.^41,44−47^ The thin film’s structural phase transition is monitored by temperature-dependent Raman spectroscopy (Figure 2c). Existing Raman peaks associated with the VO_2_ (M) are expected to disappear above the transition temperature, i.e., when the film changes to the VO_2_ (R) phase.^45^ During the heating process, the intensity of all Raman peaks gradually decreases with increasing temperature. Above 65 °C up to the highest test temperature, *T*_max_R_ = 103.4 °C all peaks belonging to VO_2_ disappear, except the peaks at about 144.5 and 520.0 cm^–1^. The peak at 520.0 cm^–1^ belongs to the silicon substrate, and as expected, a linear redshift of this peak is observed as the temperature increases (Figure S5a). The peak at 144.5 cm^–1^ is associated with the VO_2_ (M) and experiences a redshift below the transition temperature, while a significant blueshift is followed above the transition temperature, as displayed in Figure 2d. Noticeably, other VO_2_ peaks also show a similar redshift as shown in Figure S5b, c before disappearance. During the subsequent cooling process, the peaks reappear at around ∼60 °C, and gradually increase in intensity with decreasing temperature. A similar pattern has been reported for single crystal VO_2_.^48,49^ The temperature-dependent redshift can be explained by thermal lattice expansion, which results in a decrease in the phonon frequency. However, the arising blueshift at the transition temperature points to an increase in phonon frequency, which can be attributed to a lattice contraction.^46^ Such a lattice contraction is associated with the phase transition between the VO_2_ (M) and VO_2_ (R) phases, which is consistent with the *in situ* GIXRD results. Upon heating, the 144.5 cm^–1^ peak’s intensity defined over the peak area also experiences an obvious decrease due to the IMT (Figure 2e). This observed behavior of the ALD-deposited VO_2_ films is consistent with previous reports on strain studies of an epitaxial VO_2_ film^41^ and single-crystal nanobeams.^49^ Additionally, the overlapping curves from multiple temperature-dependent resistance tests on the same film sample, along with consistent Raman spectra before and after cyclic testing, indicate that the synthesized VO_2_ exhibits excellent reversible switching durability in terms of both performance and structural integrity (Figure S6).

### Optical Properties of the VO_2_ Thin Film

**Figure 3:**
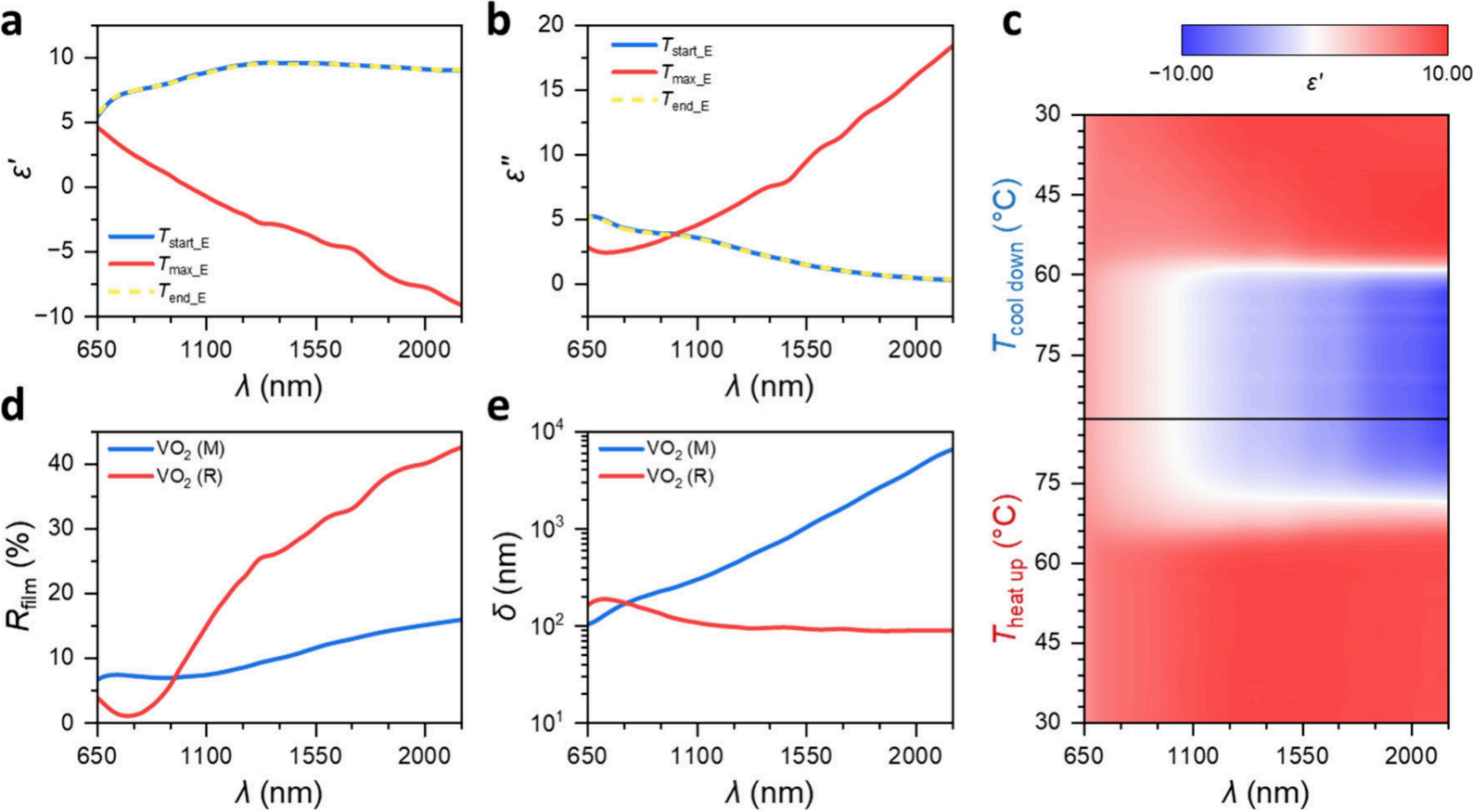
Optical characterization of ALD-deposited VO_2_ thin film. (a) The real permittivity ε′ and (b) the imaginary permittivity ε′ at the start (*T*_start_E_), maximum (*T*_max_E_), and end (*T*_end_E_) temperatures during a continuous heating/cooling measurement obtained from spectroscopic ellipsometry as a function of wavelength λ. Note that the lines for *T*_start_E_ and *T*_end_E_, plotted as a solid blue line and a dashed yellow line in (a) and (b), overlap. This match indicates a fully reversible change in the optical properties of the film. (c) Colormap of ε′ versus wavelength λ and temperature. (d) The film reflectance *R*_film_ converted from ε′ and ε″, taking the temperature at *T*_start_E_ as VO_2_ (M), and at *T*_max_E_ as VO_2_ (R). (e) Skin depth δ for the VO_2_ (M) and VO_2_ (R) phases plotted logarithmically. The skin depth increases significantly toward long wavelengths in the VO_2_ (M) phase while remaining constant in the VO_2_ (R) phase.

To better understand the optical properties of the ALD-deposited VO_2_, *in situ* temperature-dependent spectroscopic ellipsometry is used to characterize the change in complex permittivity ε̃(λ) = ε′ + *iε*″ of the thin film on the Si substrate over the wavelength λ during the IMT, where ε′ and ε″ are the real part and the imaginary part of the complex permittivity ε̃(λ), respectively. This measurement starts at room temperature *T*_start_E_ = 25.7 °C, heats to a temperature *T*_max_E_ = 88.4 °C, that is above the transition temperature, and then cools back to near room temperature *T*_end_E_ = 27.8 °C. Both ε′ (Figure 3a) and ε″ (Figure 3b) reveal distinct differences between the low temperature (*T*_start_E_, *T*_end_E_) and the high temperature (*T*_max_E_) states. Note, the typical value of ε′ of a metal is negative, while that of a dielectric exceeds 1. At low temperatures, ε′ of the VO_2_ thin film indicates optically lossy dielectric behavior over the entire measured wavelength range. Whereas, at elevated temperatures, ε′ remains above 1 only for wavelengths shorter than 1 μm, indicating that the VO_2_ thin film, despite the change in value, retains lossy dielectric characteristics in this wavelength region. In contrast, for wavelengths longer than 1 μm, ε′ becomes negative, revealing metallic optical characteristics. Limiting the discussion to this long wavelength range, these results demonstrate that the crystalline phase transition induces a pronounced change in optical properties. This observation is in good agreement with other reported VO_2_ thin films produced by sputtering or sol–gel.^20,50−52^ Specifically, the film begins its optical transition to the metallic VO_2_ (R) phase at ∼70 °C during heating and transitions back to the dielectric VO_2_ (M) phase at ∼60 °C during the following cooldown process, as shown in the temperature-dependent colormaps of ε′ and ε″ in Figure 3c and Figure S7a, respectively. In addition, similar to the resistive and structural transitions, the optical transition also presents a hysteresis in the heating and cooling process. The film reflectance *R*_film_ can be mathematically obtained with the transfer-matrix method from the measured ε̃(λ) (Figure S7b).^53^ All the colormaps show a distinct IMT between the VO_2_ (M) and VO_2_ (R) phases during the heating and cooling processes. When *R*_film_ is plotted as line curves, the optical effect of the IMT on the reflection can be clearly seen (Figure 3d). The skin depth δ, which is the distance an electromagnetic wave travels through a material before its power is reduced to 1/*e*^2^, is used to explain the optical attenuation characteristics of VO_2_. It can be calculated using the equation  derived from the relation *ñ*(λ) = √ε̃(λ), where *ñ*(λ) = *n* + *ik* is the complex refractive index, and *n* and *k* are its real and imaginary part, respectively.^54^ The skin depth of the low temperature VO_2_ (M) phase increases towards longer λ values, as shown in Figure 3e. In contrast, the skin depth is relatively constant in the high temperature VO_2_ (R) phase and remains below 200 nm over the entire λ range considered. Moreover, the detailed *n* and *k* also reveal a distinct difference between the VO_2_ (M) and VO_2_ (R) phases (Figure S8).

### Fabrication of a VO_2_ IO PhC

**Figure 4:**
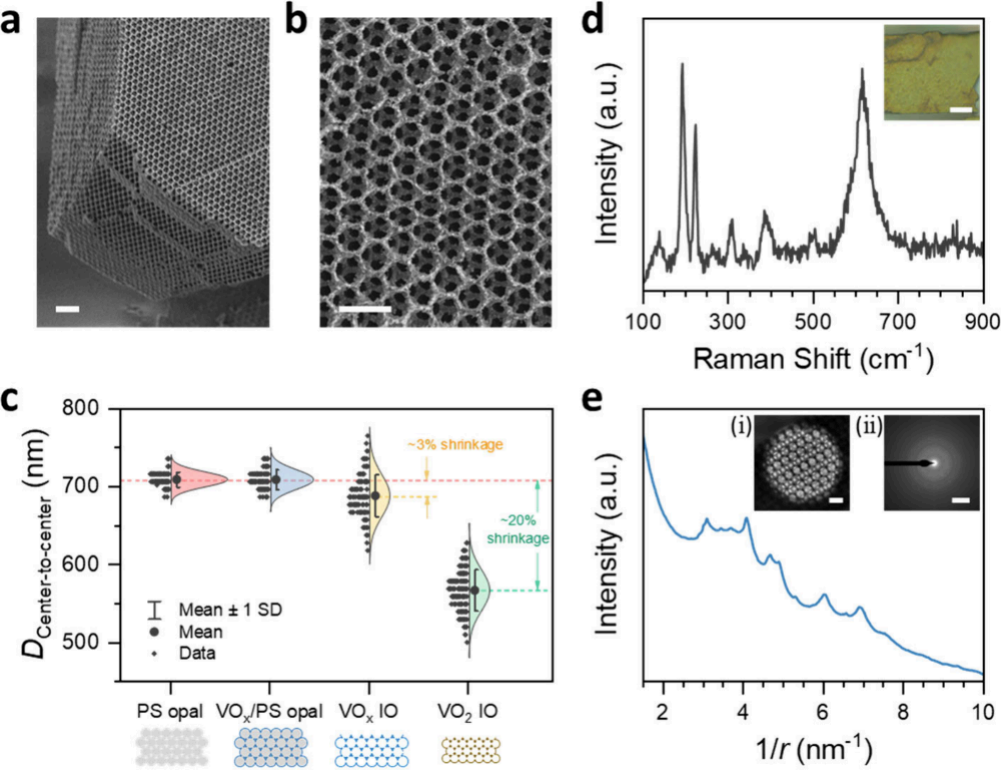
VO_2_ IO PhC fabrication. SEM images of the VO_2_ IO (a) from the side view at low magnification and (b) from the top view at higher magnification. (c) Statistics of center-to-center distance, *D*_Center-to-center_, between adjacent PS spheres or macropores during the IO fabrication process: (I) Pristine PS opal template, (II) VO_*x*_ coated PS opal template (VO_*x*_/PS opal), (III) VO_x_ IO, and (IV) VO_2_ IO PhC. The *D*_Center-to-center_ is measured using ImageJ software on the corresponding SEM images. The distribution curves were normalized according to the area under the curve. (d) Raman spectrum for the VO_2_ IO. Inset: optical image of the VO_2_ IO. (e) Radially averaged profiles of the IO lamella’s electron diffraction ring using TEM before and after a heating/cooling cycle showing an unchanged VO_2_ phase. Inset (i): TEM image for the VO_2_ IO lamella. Inset (ii): corresponding electron diffraction pattern. Scale bars for (a), (b), (d-inset), (e-inset (i)), and (e-inset (ii)) are 2 μm,1 μm, 20 μm, 500 nm, and 5 nm^–1^, respectively.

All of the above results, coupled with the inherent conformality and Angstrom-level controllability of ALD coatings, render the ALD-based VO_2_ synthesis process a suitable candidate for fabricating switchable 3D photonic structures. By exploiting the pronounced reflectivity switching of VO_2_ in the NIR region induced by temperature changes, alongside the presence of a PBG of the PhC structure, we devised a controllable PhC with an IO structure. This PhC allows for active control of the PBGs through temperature switching, combining a narrow-band NIR reflector—determined by the PhC’s PBG, and a broad-band absorber given by the material’s properties. The fabrication details are described in the Materials and Methods section. The successfully fabricated VO_2_ IO PhC is shown in Figure 4. The VO_2_ IO exhibits a 3D periodic and ordered multilayer porous structure throughout, from top to bottom, as presented in Figure 4a. This homogeneity demonstrates that the ALD coating is conformal within the opal structure. Figure 4b further highlights the high uniformity and regularity of the pore arrangement. Additionally, the stability of the IO structure is conserved upon heat treatment and crystallization. However, compared to the pristine PS opal template, the VO_2_ IO experiences significant shrinkage (Figure S9). The center-to-center distance, *D*_Center-to-center_, between adjacent PS spheres or macropores at different stages in the fabrication process is statistically compared, as shown in Figure 4c. The *D*_Center-to-center_ between adjacent PS spheres in the pristine template is 708.7 ± 9.66 nm. After VO_x_ ALD coating, the *D*_Center-to-center_ between adjacent VO_x_-coated PS spheres is 708.6 ± 12.67 nm, remaining almost unchanged. But after removal of the PS template performed at 390 °C in vacuum (Materials and Methods, Figure S10), the *D*_Center-to-center_ between adjacent macropores decreases to 687.8 ± 27.05 nm, accounting for a shrinkage of approximately 3%. During the subsequent annealing process to adjust the IO shell composition from VO_x_ to VO_2_, the *D*_Center-to-center_ between adjacent macropores further shrinks significantly to 566.7 ± 24.45 nm, resulting in a further shrinkage of approximately 17.6%. Overall, compared to the pristine PS opal template, the VO_2_ IO shrinks by approximately 20%. This observed shrinkage is about double the value previously observed for zirconia, titania, and mullite IOs.^55−57^ However, in our work, we expect that not only crystallization would occur but also phase transitions between different vanadium oxide phases with an associated shrinkage of the unit cell. Thus, the hereby observed shrinkage has the contribution from the thermal shrinkage related to crystallization as observed in other reports,^55−57^ as well as the material phase change. In addition to the 3D morphology change in macropore size, the material itself undergoes a complex process and is successfully converted into VO_2_ by a final postdeposition annealing step. The Raman spectrum of such a prepared IO is highly consistent with that of the VO_2_ film as shown in Figure 4d, which indicates that the skeleton of IO is indeed pure VO_2_ with the V oxidation state +4. Thus, the additional shrinkage from the calcined VO_x_ IO to the VO_2_ IO is attributed to the change in oxidation state and not to solely crystallization. However, to fully understand the volume shrinkage, it is necessary to determine the precise VO_x_ stoichiometry in all manufacturing stages. Additionally, a piece of an IO lamella (Figure S11) is prepared to further study the VO_2_ IO structure by using transmission electron microscopy (TEM). The radial distribution profile of the IO lamella’s electron diffraction ring matches well with the same PDF card used for thin film XRD matching (Figure 4e, Figure S12). This agreement also confirms the successful fabrication of an IO made out of VO_2_. In addition, the composition and microstructure of the VO_2_ IO lamella remains unchanged after being heated to 100 °C and then cooled back to room temperature (Figure S12).

### Switchable Photonics Characterization

**Figure 5:**
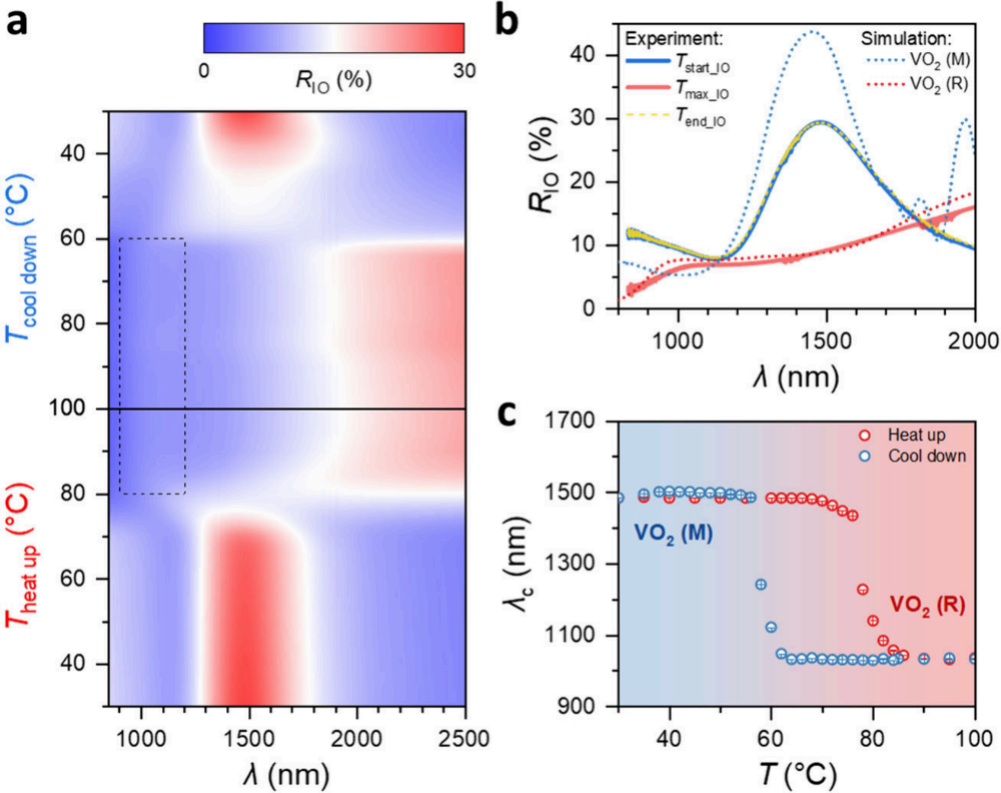
Switchable PBG of VO_2_ IO PhC. (a) Reflection spectra *R*_IO_ during a heat-up and following cool-down measurement. (b) Experimental *R*_IO_ and simulated *R*_IO_ at high and low temperatures. The experimental results for *T*_start_IO_ and *T*_end_IO_ overlap. The small peak in the 900–1200 nm region of the curve marked as *T*_max_IO_ in (b) is highlighted in the roughly corresponding region with a black dashed box in (a). The oscillation in the simulated spectrum of the VO_2_ (M) phase results from Fabry–Perot interference. (c) The Bragg peak position λ_c_ as a function of temperature, showing a switch function adapted to the external temperature. The Bragg peak position λ_c_ shifts from about 1.49 μm below the IMT transition temperature to about 1.03 μm above the IMT transition temperature.

Detailed reflection spectra *R*_IO_ of the VO_2_ IO PhC are recorded during *in situ* heating and cooling. At low temperatures, when the VO_2_ is in the dielectric state (M), e.g., at *T*_start_IO_ = 30 °C, a distinct PBG is identified in the NIR region with the center position of the Bragg peak λ_c_ ≈ 1.49 μm, as shown in temperature-dependent *R*_IO_ in Figure 5a. This PBG is induced by the IO structure consisting of face-centered cubic stacked sphere-shaped air cavities embedded in the VO_2_ backbone, forming a periodic permittivity alteration. When the temperature rises above 80 °C, this peak in the NIR wavelength range vanishes due to the IMT from VO_2_ (M) into VO_2_ (R), which is the metallic phase, and a small peak at around λ_c_ ≈ 1.03 μm is observed. The occurrence of this peak can be attributed to the transition in optical parameters due to the IMT, as estimated in Note 1. Note that this peak is not as pronounced as that observed in the dielectric VO_2_ (M) phase since the real part of the refractive index, *n*, of VO_2_ in the VO_2_ (R) phase is considerably lower than that in the VO_2_ (M) phase, while the imaginary part, *k*, is higher (Figure S8). This fact implies that the reflection at each stacking layer is small. Hence, only a fraction of the incident light power is reflected, whereas the majority penetrates the structure and is absorbed. Moreover, due to increased absorption, the peak spectrally widens, making it less pronounced. In addition, similar to the *R*_film_ of the VO_2_ (R) in Figure 3, the *R*_IO_ becomes broadband toward longer λ as the temperature increases. During the subsequent cooling process, the Bragg peak λ_c_ ≈ 1.49 μm reappears as the dielectric state is restored and the photonic structure reverts to being a narrow-band NIR reflector. Figure 5b shows in more detail the reflectivity curves corresponding to the starting temperature *T*_start_IO_, maximum temperature *T*_max_IO_, and end temperature *T*_end_IO_ in this experiment. In addition, the electromagnetic simulation results for the VO_2_ IO PhC in the dielectric VO_2_ (M) phase (corresponding to the low temperatures, *T*_start_IO_, and *T*_end_IO_) and the metallic VO_2_ (R) phase (corresponding to high temperature, *T*_max_IO_) are compared with the experimental reflectance spectra, showing a good agreement, particularly in the general position and relative intensity of the Bragg peaks. Minor deviations between simulation and experiment mainly stem from the fact that the macropore size has a certain distribution, the existence of cracks, and the roughening of the surface of the VO_2_ shell due to the annealing, resulting in diffuse scattering beyond the microscope’s aperture. The Bragg peak’s position λ_c_ during the temperature-dependent measurement from Figure 5a is presented in processed form in Figure 5c, revealing a reversible switch with external temperature. Specifically, when VO_2_ is in the VO_2_ (M) phase (low temperature), only the PBG near 1.49 μm in the IO exists. But it switches off at high temperatures when the VO_2_ is in the VO_2_ (R) phase. The situation of the PBG near 1.03 μm is just the opposite. Similarly, there is a hysteresis in this switching, which is consistent with the other fixed positions of *R*_IO_ in Figure S13. Besides, the variation of *R*_IO_ with temperature also exhibits a hysteresis, which is wider than that observed in thin films (Figure S13). Earlier reports show that the hysteresis of VO_2_ in thin films can be altered by introducing either tensile or compressive strain, resulting in a narrower or wider hysteresis, respectively.^58,59^ The 20% shrinkage observed during synthesis likely introduced residual stresses, which may differ from those of the thin film due to the different underlying substrates. While the thin film is prepared on top of a silicon substrate, the IO is assembled on top of a glass microscope glass substrate. Moreover, they have different thicknesses of around 0.5 mm for the Si wafer against 1 mm of the glass, likely leading to a lower surface temperature of the IO during measurement in comparison to the thin film since the heating plate heats them from underneath the substrate. All combined results in a wider IMT hysteresis in the VO_2_ IO.

## Conclusions

We successfully prepared high-quality polycrystalline VO_2_ thin films via ALD combined with a postdeposition annealing treatment. Furthermore, the synthesis route was then adapted to conformally coat opaline PS templates, resulting in the successful fabrication of switchable VO_2_ IO PhCs. The VO_2_ thin films demonstrated an IMT in structural, electrical, and optical properties at about 60–70 °C, similar to previously reported VO_2_ thin films obtained by other methods, e.g., magnetron sputtering, molecular beam epitaxy, and sol–gel. The integration of the VO_2_’s IMT properties with the IO PhCs structure opens active capabilities other than conventional photonics. Our VO_2_ IO PhC exhibits a reversible switching between two different bandgaps at nearly 1.49 and 1.03 μm at low-temperature dielectric state and high-temperature metallic state, respectively. This work provides a convincing example of the integration of ALD-based VO_2_ coating and devices with complex structures, holding significant promise for further unleashing the potential of VO_2_ as the route herein described can be applied to other low-temperature substrates such as 3D-printed polymers.

## Materials and Methods

### Thin Film Fabrication

The VO_2_ thin film fabrication process involves two steps. Initially, a nonstoichiometric VO_x_ layer was deposited via thermal ALD in exposure mode, utilizing a modified Savannah100 reactor (Cambridge Nanotech). The deposition was conducted at 95 °C with 30 sccm (standard cubic centimeter per minute) nitrogen as carrier gas and purge gas. The working pressure during deposition was approximately 1.5 Torr. Various substrates including SiO_2_/Si wafers and pure polished Si wafers (SIEGERT WAFER GmbH) were employed for coating. The precursors, deionized water and tetrakis(dimethylamino)vanadium(IV) (TDMAV, Strem Chemicals, Inc., USA) were utilized as oxygen and vanadium sources, respectively. The combination of “precursor pulse time/exposure time/purge time” was set as 0.1/15.0/60.0 s for deionized water and 1.0/15.0/60.0 s for TDMAV. Subsequently, the VO_x_ film was annealed at 425 °C in a vacuum oven (MC050, ANNEALsys) at 0.1 Torr for 10 min within a controlled atmosphere (10 sccm O_2_, 100 sccm N_2_).

### VO_2_ IO PhC Fabrication

The vertical self-assembly of the 757 nm diameter polystyrene beads (Microparticles GmbH) into an opal structure with FCC stacking followed a previously published procedure.^57^ Next, the template was placed in the ALD chamber and coated by VO_x_ at 95 °C with a prolonged exposure time and purge time, i.e., 0.1/60.0/90.0 and 1.0/60.0/90.0 s, for deionized water and TDMAV, respectively. These parameters were set to enhance precursor penetration into the voids within the template, based on our previous studies on titania and mullite.^48,60^ Following this, a portion of the top layer of the coated sample was etched by using a reactive ion etching system (SENTECH SI 500) via Ar ion sputtering. Afterward, template removal, coupled with a postdeposition annealing process, was conducted in the vacuum oven (MC050, ANNEALsys) to achieve the VO_2_ phase in the 3D structure of the IO PhC. The annealing cycle consisted of two steps: (1) Controlled PS template removal to avoid potential phase changes from the VO_x_ phase to the V_2_O_5_ phase or distortion of the 3D structure in the second step. Specifically, the template removal was performed at 390 °C, near the polystyrene vaporization point, with a 1 °C/min heating rate in a vacuum of 0.01 Torr and maintained for 6 h. (2) Then the temperature was tuned to 425 °C, 200 sccm N_2_, and 10 sccm O_2_ were input, and the pressure was maintained at 0.1 Torr for 20 min for the subsequent postdeposition annealing process to achieve VO_2_ phase.

### Thin Film Characterization

Water-diluted HCl (20:1) was used to etch the as-deposited film to prepare a step-edge for thickness measurements by a profilometer (DektakXT, Bruker). Atomic force microscopy (AFM, Dimension ICON, Bruker) was applied to determine the thin film’s roughness. Electrical characterization was carried out in a Physical Property Measurement System (VersaLab, Quantum Design). The crystalline phases of the thin films were determined by grazing incidence X-ray diffraction analysis (Bruker AXS D8 Advance, Bruker) using Cu Kα radiation. The incident angle was 0.5°, with the range, step size, and step time set to 26.5 to 29.0°, 0.01°, and 2 s for the *in situ* measurements and 10° to 60°, 0.01°, and 2 s for the *ex situ* measurements. The *in situ* measurements were performed by using a high-precision hot stage (DHS 900, Anton Paar) and a heating controller (TCU 150, Anton Paar). Heating and cooling cycles were performed from 25 °C until 90 °C with increments of 5 °C per measurement. Before data collection, samples were allowed to equalize the temperature for 5 min at each temperature data point. To ensure that no other phase change occurred due to the heating/cooling cycles, the samples were analyzed *ex situ* at room temperature (25 °C) before and after the *in situ* measurements. Raman measurements were performed using Renishaw inVia Raman Microscope equipped with a 20× objective, 1200 grooves/mm grating, and an excitation wavelength of 532 nm at a laser power of 1.7 mW. The Raman system incorporated a homemade temperature controller comprising a thermoelectric power generator Peltier module (SP1848–27145, VGEBY), a copper water cooling unit, and a voltage source. The optical measurements of the thin films were performed with a spectroscopic ellipsometer (SE-2000, SEMILAB) equipped with a heating chamber (Linkam). All measurements were performed at an incidence angle of 65° within the wavelength range of 650 to 2150 nm. The sample consisted of a 138.6 nm VO_2_ layer on a 2 nm native SiO_2_ layer on a Si substrate. The sample was heated in an open chamber in an air atmosphere to mitigate the influence of windows during data collection, reaching temperatures of up to 88 °C in increments of approximately 2 °C with a 120 s holding time before starting the spectroscopy ellipsometry measurement. Temperature readings were calibrated by using a thermoelement (NiCr-Ni) attached to the upper surface of the sample. The ε̃(λ) of the VO_2_ layer was obtained from the measured ellipsometry angles (Psi and Delta) by mathematical inversion method^61^ and afterward checked for consistency with Kramers–Kronig relations. Both methods are implemented by Semilab in their Spectroscopic Ellipsometry Analyzer (SEA) software.

### Characterizations of VO_2_ IO PhC

SEM images were taken via scanning electron microscopy (SEM, Supra 55 VP, Zeiss). FIB-preparation and transfer of the TEM-lamella to the *in situ* MEMS chip were done with a Thermo Fisher Helios G3 UC according to the standard lift-out procedure. Contacting of the lamella to the MEMS chip was performed with beam-induced platinum deposition. The *in situ* experiments were performed in a Thermo Fisher Talos F200X transmission electron microscope equipped with a Thermo Fisher NanoEx-i/v *in situ* TEM sample holder. TEM micrographs were recorded in BF-TEM mode at 200 kV and 10 nA beam current. SAED patterns were recorded at a camera length of 840 mm. Intensity profiles were extracted using Velox TEM software (Thermo Fisher). Control of Temperature and heat rate was done with NanoEx-control software. The reflection spectra of the IO PhC samples were recorded using an FT-IR spectrometer Vertex 70 combined with a microscope Hyperion 2000 (Bruker Optics) and a Linkam Heating chamber TS1500. During heating, increments of 5 °C were applied for each measurement within the temperature ranges of 20 to 60 °C and 90 to 105 °C. Additionally, a smaller increment of 2 °C was applied near the IMT temperature, specifically within the range of 60 to 86 °C. In the cooling stage, increments of 5 °C were set within the temperature ranges of 105 to 85 °C and 84 to 20 °C. Furthermore, a smaller increment of 2 °C was applied within the temperature range of 60 to 38 °C.

### Electromagnetic Simulation

The simulations were performed using a Frequency Domain Solver with hexagonal unit cell boundary conditions from the CST Studio Suite (Dassault Systèmes). The simulation model (see Figure S14) is designed as follows: there are six consecutive layers of spheres of 566 nm diameter, which are assembled into an FCC lattice with the (111)-plane parallel to the surface. For VO_2_, we applied the permittivity function obtained from the ellipsometry measurements of the thin film. Below the spheres, a glass substrate with ε = 2.25 was considered. The background is air.

## Data Availability

The data supporting the findings of this work are available within the article and its Supporting Information files. All other relevant data supporting the findings of this study are available from the corresponding author on request.

## Supporting Information Available

Additional experimental details, methods, and simulation details, including data figures, experimental setup, and explanation (PDF)
