# Supplementary material for: Switchable 3D Photonic Crystals Based on the Insulator-to-Metal Transition in VO2
Source: ACS Appl Mater Interfaces. 2024 Dec 2;16(49):67106–15. doi: 10.1021/acsami.4c13789 (PMC11647753; doi:10.1021/acsami.4c13789)
Supplement: Supplementary file 1 — am4c13789_si_001.pdf [file am4c13789_si_001.pdf]

Supporting Information

Switchable 3D Photonic Crystals Based on the Insulator-to-Metal Transition in VO_2

Jun Peng[1], Julia Brandt[2], Maurice Pfeiffer[2], Laura G. Maragno[3], Tobias Krekeler[4], Nithin T. James[3], Julius Henf[2], Christian Heyn[1], Martin Ritter[4], Manfred Eich[2,5], Alexander Yu. Petrov[2,5], Kaline P. Furlan[3], Robert H. Blick[1,6], Robert Zierold[1].*

1 Center for Hybrid Nanostructures, Universität Hamburg, Luruper Chaussee 149, 22607 Hamburg, Germany

2 Institute of Optical and Electronic Materials, Hamburg University of Technology, 21073, Hamburg, Germany

3 Integrated Ceramic-Based Materials Systems Group, Hamburg University of Technology, 21073, Hamburg, Germany

4 Betriebseinheit Elektronenmikroskopie, Hamburg University of Technology, 21073, Hamburg, Germany

5 Institute of Functional Materials for Sustainability, Helmholtz-Zentrum Hereon, 21502 Geesthacht, Germany

6 Deutsches Elektronen-Synchrotron (DESY), Notkestr. 85, 22607 Hamburg, Germany

*Corresponding Author: Robert Zierold, rzierold@physnet.uni-hamburg.de

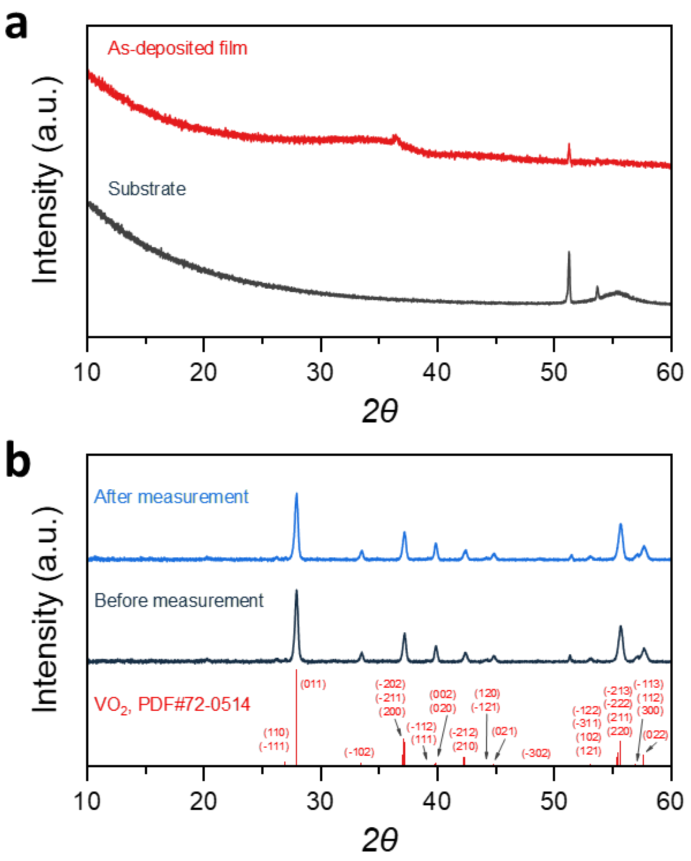

Figure S1. Comparisons of *ex situ* GIXRD measurements. (a) Comparison of the as-deposited film and the substrate's GIXRD signal. In the as-deposited film, the two peaks at $2\theta \approx 51.2°$ and 53.6° come from the substrate. The weak peak at $2\theta \approx 36.5°$ might be contributed by many vanadium oxides compounds such as V_2O_3, V_5O_9, V_6O_{13}, VO_2. (b) The full measurements of an annealed thin film before and after the *in situ* temperature-dependent measurements showing no alteration in the main phase due to the heating/cooling cycle. The dominant compound in the annealed film is VO_2 (PDF#72-0514, $P2_1/c$).

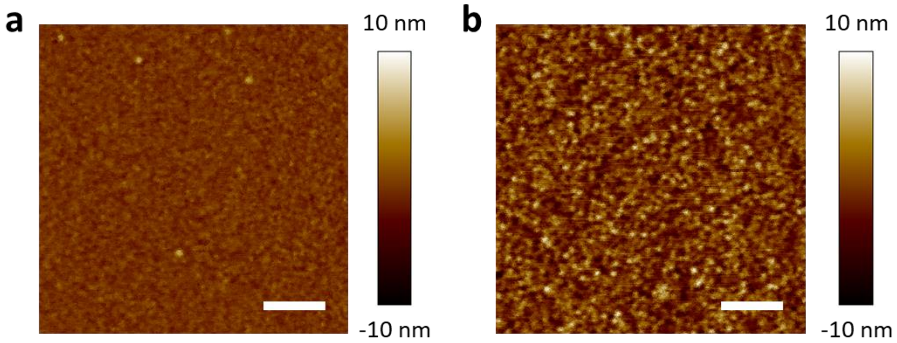

Figure S2. The surface morphology comparison for the same sample before and after post-deposition annealing using AFM. (a) The initial as-deposited VO_x film with a thickness of 33.6 nm is smooth and dense, characterized by a surface roughness (R_a) of 0.57 nm. (b) Subsequent to annealing, the VO_x film underwent crystallization, transforming into a crystallized VO_2 film, accompanied by an increase in surface roughness to 1.63 nm. Scale bars for (a), (b) are all 1 μm.

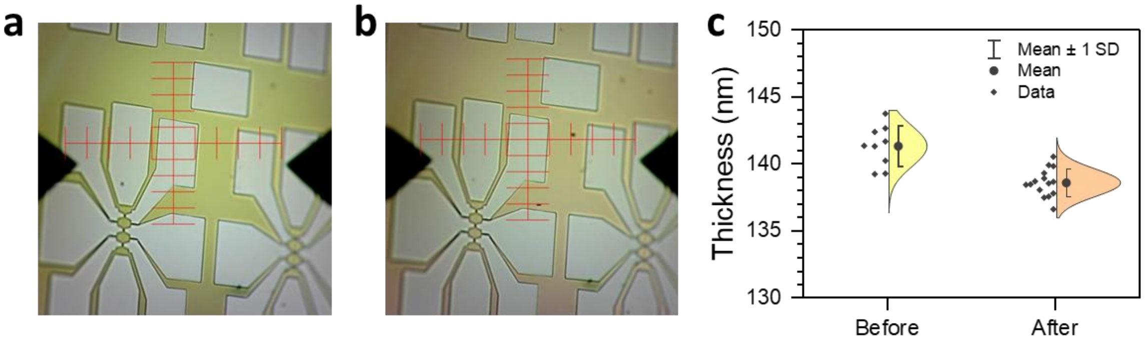

Figure S3. Optical microscope photos of the same thin film sample at the same position (a) before and (b) after annealing. The specific pattern on the sample has no meaning, it is generated during the process of constructing the step height for the step meter to measure. First, the pattern is defined on the VO_x film through a standard photolithography process, then the film within the developed area is etched with a chromium etching solution, followed by a clean process using acetone, isopropanol, and deionized water, resulting in a VO_x film with steps. These photos are derived from a file profilometer, and the length of each small transverse grid in the cross ruler is approximately 150 μm. After annealing, the color of the film becomes darker due to slight change in stoichiometry to the VO_2 phase. (c) Results of multiple thickness measurements on the same sample before and after annealing. Annealing reduces the thickness of the film from 141.3 ± 1.52 nm to 138.6 ± 1.03 nm, a decrease of approximately 2%.

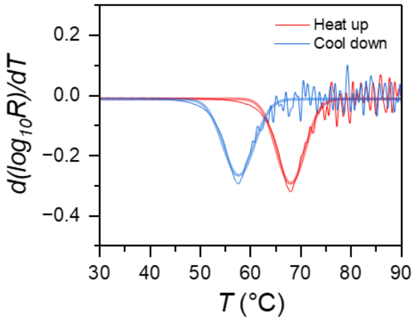

Figure S4. The corresponding Gaussian fitting of the derivation of the logarithmic *R* hysteretic curve in Figure 1d to determine the transition temperature and hysteresis. The transition temperature during heating is 68.0 °C, while during cooling is 57.7 °C, with a hysteresis of 10.3 °C. Taking the average value of the IMT temperatures during the heating and cooling processes as the IMT temperature of the sample, the IMT temperatures of the film during the electrical measurement is 62.85 °C.

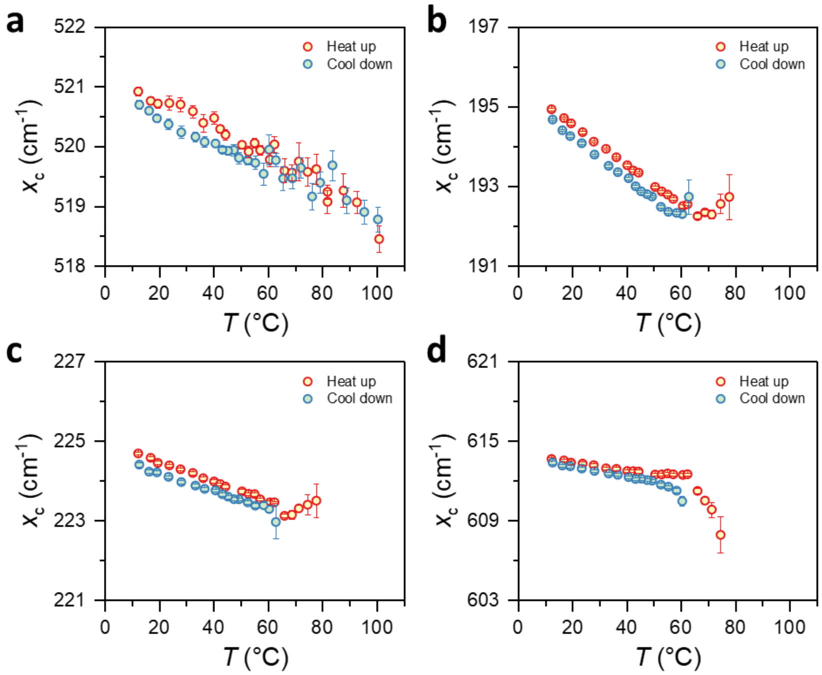

Figure S5. The shift of Raman peak position x_c peaks during the temperature-dependent Raman characterization in Figure 2c. (a) The Si signal from the substrate. It redshifts due to the lattice expansion with temperature. The three typical VO_2 signals at near (b) 194 cm^{-1}, (c) 244 cm^{-1}, and (d) 612 cm^{-1} at room temperature. The two characteristic peaks at near 194 cm^{-1} and 244 cm^{-1} both with A_g symmetry are assigned to V–V vibration modes, and the high-frequency peak at near 612 cm^{-1} with A_g symmetry relates to the V–O vibrations.[45] The three peaks show a slight redshift between RT and near 60 °C. However, the peaks near 194 cm^{-1} and 244 cm^{-1} show a significant blue shift at temperatures above 60 °C, while the peak near 630 cm^{-1} exhibits more pronounced redshift. After about 80 °C, all peaks disappear. These strong changes occur near the IMT temperature.

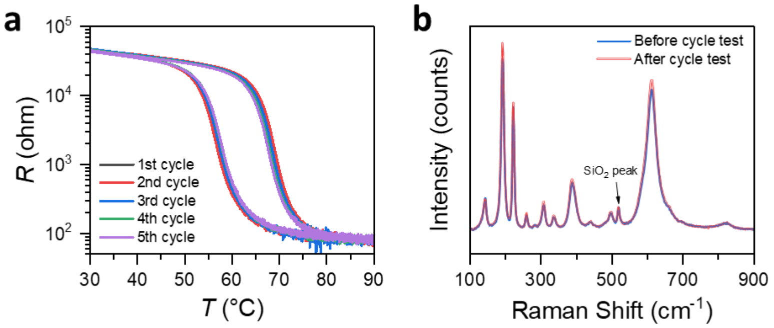

Figure S6. Reversible switching durability test of the VO_2 thin film. (a) Five consecutive temperature-dependent resistance tests. (b) Raman spectra comparison before and after the temperature-dependent resistance tests. The overlap character in both tests confirms the superior reversible switching properties of the ALD-assisted synthesized VO_2.

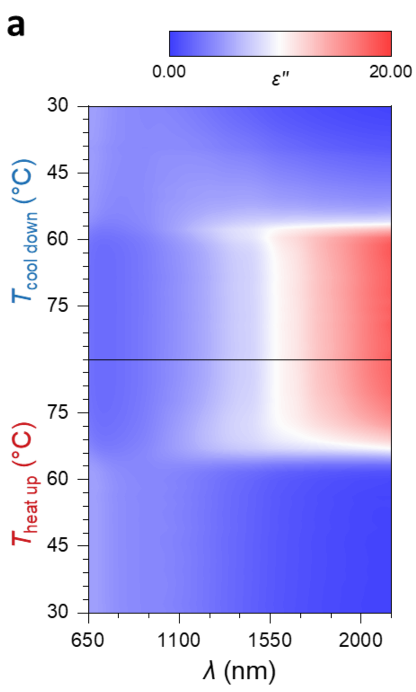

Figure S7. (a) Colormap of the temperature-dependent imaginary permittivity ε''. (b) Colormap of the film reflectance R_{film} converted mathematically from ε' and ε'' with the transfer-matrix method and plotted as a function of wavelength and temperature.

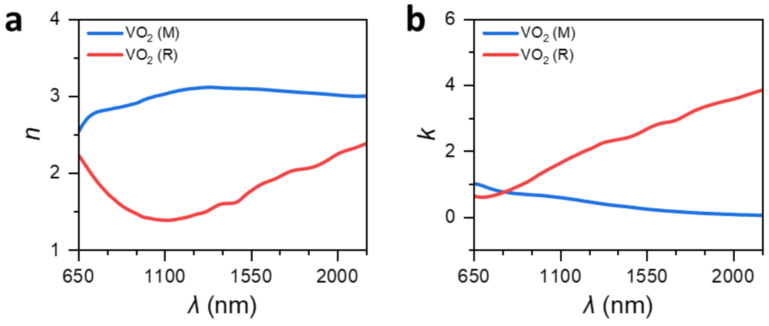

Figure S8. Complex refractive index of the VO_2 thin film. (a) *n*. (b) *k*.

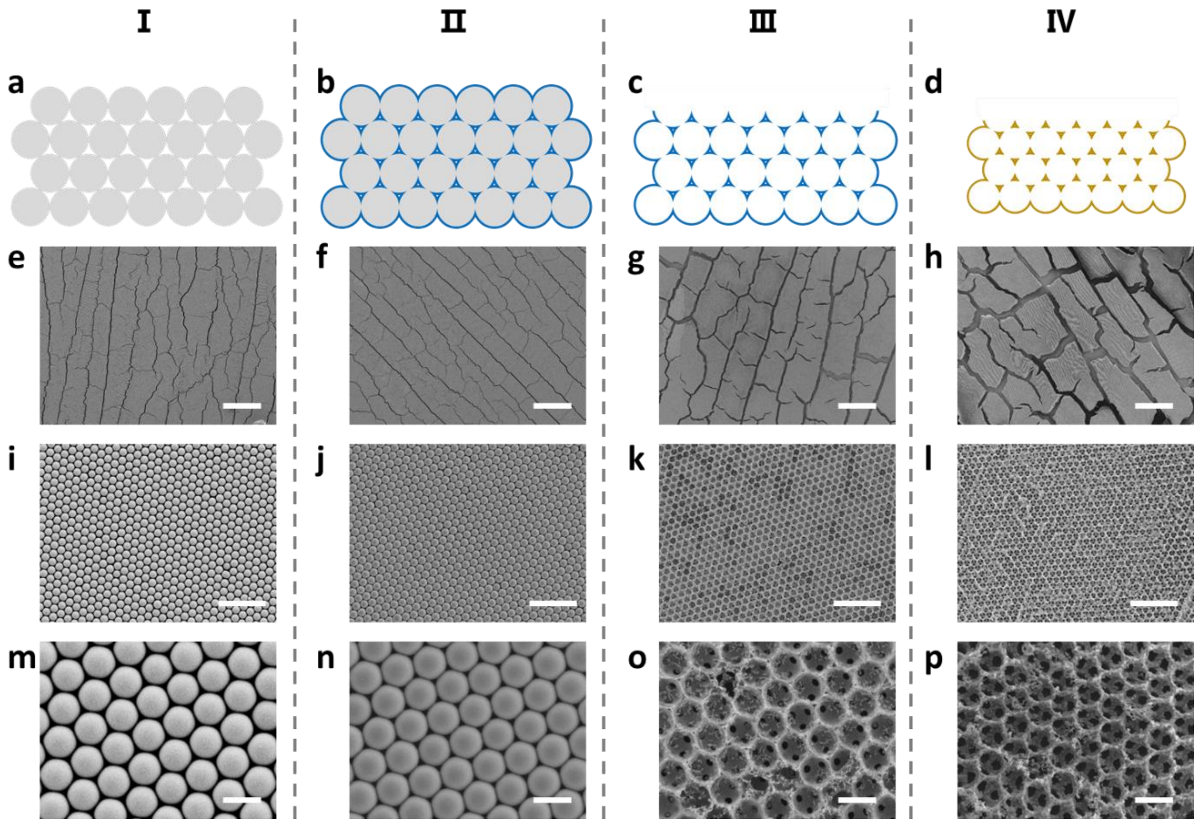

Figure S9. Shrinkage of the sample during preparation. Pristine PS opal template (column I), VO_x coated PS template (column II), VO_x IO (column III), and VO_2 IO PhC (column IV). (a~d) the side view sketch of the opal or IO layer at stages I, II, III, and IV. (e~h) SEM images for the samples at stages I, II, III, and IV. The scale bars are all 80 µm. (i~l) Zoomed-in SEM images for the samples at stages I, II, III, and IV. The scale bars are all 4 µm. (m~p) Further zoomed-in SEM images for the samples at stages I, II, III, and IV. The scale bars are all 800 nm. In (g) and (h), the gaps between opal blocks increase continuously compared to those in (e) and (f), and traces of the original pure PS template's location can even be seen in some large gaps. This maybe the results of the shrinkage.

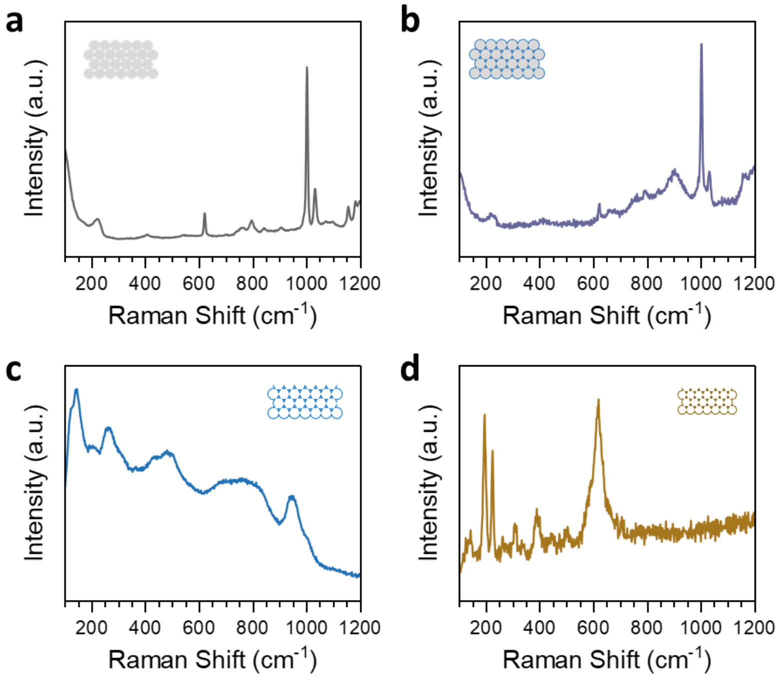

Figure S10. Raman spectra of the main steps in the VO_2 IO process. (a) Pristine PS opal template. (b) VO_x coated PS template. After ALD deposition of VO_x, the curve is still dominated by PS signals, but a new peak belonging to vanadium oxide compounds appears around 700 ~ 950 cm^{-1}. (c) VO_x IO. This curve does not contain the main peak of V_2O_5. (d) VO_2 IO PhC.

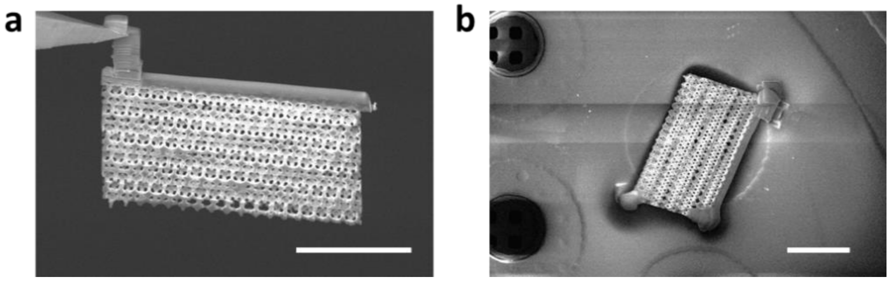

Figure S11. SEM images of the VO_2 IO lamella for the TEM characterization. Scale bars for (a), and (b) are all 5 μm.

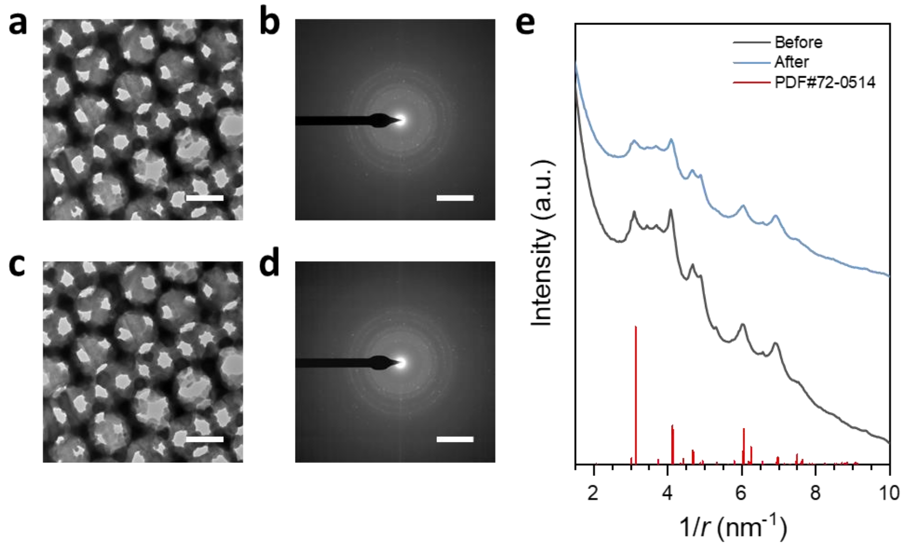

Figure S12. Detailed comparison of the TEM characterizations before and after heat-up and cool-down treatment. (a) TEM image and (b) corresponding diffraction ring pattern before heat-up treatment. (c) TEM image and (d) corresponding diffraction ring pattern after cool-down treatment for the same lamella at the same area. (e) Comparison of radial integrated diffraction patterns before and after heat-up and cool-down treatment. Peak position in the intensity profiles match well with values from PDF card (PDF#72-0514, $P2_1/c$), indicating the good structural integrity during the IMT phase transition. The faint peak near $1/r$ = 3.44 nm^{-1} can be explained with nanocrystalline gallium, which is a residue of FIB preparation of the lamella. The peak position corresponds to the (111) lattice plane distance in alpha-Ga (2.95Å). Scale bars from (a) to (d) are 500 nm, 5 nm^{-1}, 500 nm, and 5 nm^{-1}, respectively.

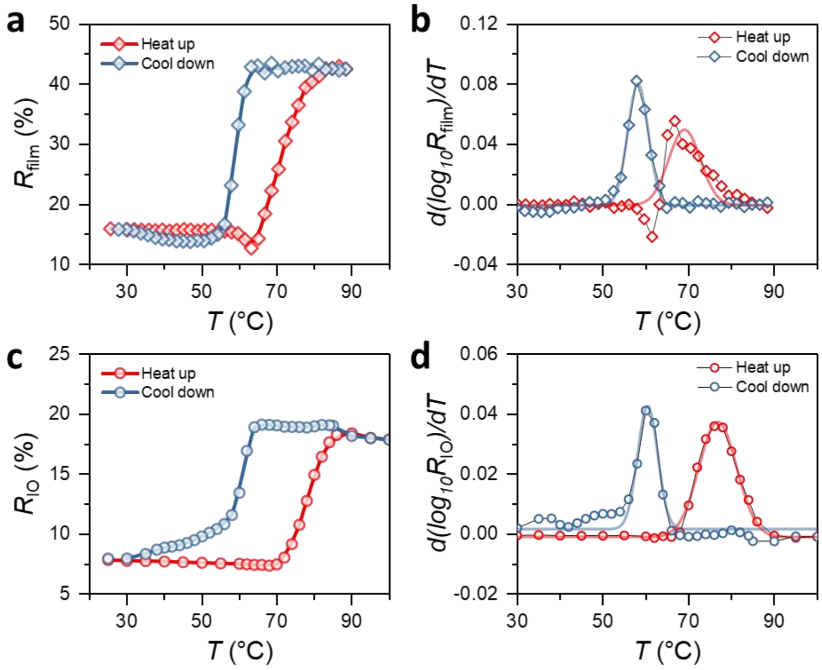

Figure S13. Comparison of the temperature-dependent reflectance between the VO_2 thin film and the VO_2 IO PhC at the wavelength of 2150 nm. (a) The VO_2 thin film reflectance R_{film} and (b) the corresponding Gaussian fitting of the derivative of the logarithmic R_{film} hysteretic curve to determine the transition temperature and hysteresis. (c) The VO_2 IO PhC reflectance R_{IO} and (d) the corresponding Gaussian fitting of the derivative of the logarithmic R_{IO} hysteretic curve to determine the transition temperature and hysteresis. This wavelength is picked because it is in the near IR region where the *R* of both thin film and IO are not affected by the structure judging from Figure 3c and Figure 5a. Remarkably, the hysteresis in the heating and cooling process is quite similar to the trend of the typical IMT on resistance as shown Figure 1d. The IO sample shows a higher transition temperature (76.8 °C) than that of the thin film sample (69.1 °C) during the heating process, while during the cooling process, the transition temperature of the IO sample (60.5°C) is comparable to that of the thin film sample (58.2°C). Taking the average value of the IMT temperatures during the heating and cooling processes as the IMT temperature of the sample

for comparison, the IMT temperatures of the film and IO samples are 63.65 °C and 68.65 °C, respectively.

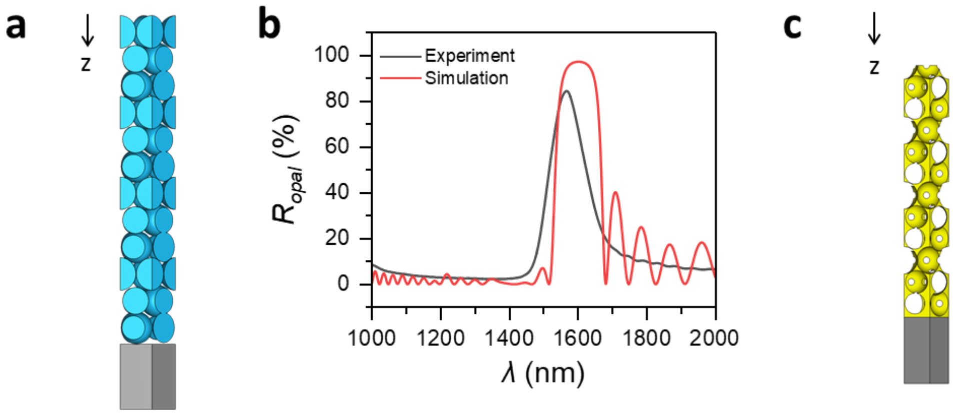

Figure S14. Simulation models. (a) Simulation model for the direct PS opal template. (b) Comparison of the opal reflection R_{opal} from the experiment and simulation. Both center positions of the peaks align well with each other. The oscillations in the simulated spectrum result from Fabry-Perot interference. The experimental spectrum does not show these oscillations due to slight variation of the opal thickness in the illuminated area. (c) The VO_2 IO PhC simulation model. The models in (a) and (c) consist of one unit cell of the FCC lattice with the (111)-plane oriented parallel to the surface. The light is incident along the z-axis.

Note 1. Estimation of the Bragg peak

Bragg's law is applied to calculate the center spectral position of the Bragg peak, $\lambda_c = 2n_{\mathrm{eff}}d_{111}$, where $d_{111} = \sqrt{\frac{2}{3}}d$ being the lattice constant along the [111]-direction in the FCC-stacked sphere-shape air within the VO_2 backbone and $n_{\mathrm{eff}} = \sqrt{fn_1^2 + (1-f)n_2^2}$ the real part of the effective refractive index, with $f = 0.2$ as the filling fraction, $n_2 = 1$ as the refractive index of air, d as the diameter of the spherical motif and n_1 is the real part of the refractive index of VO_2. The applicability of Bragg's Law is constrained to non-dispersive and lossless dielectrics. Following the ellipsometry results shown in Figure 3 and Figure S7, we approximate the real part of the refractive index of VO_2 in its dielectric phase to be $n_1 = 3.05$. For a sphere diameter of 566 nm, the Bragg peak is at 1.5 μm. The real part of the refractive index in the metallic phase is $n_1 = 1.7$. Using the same parameters as before, we calculate the spectral position of the Bragg peak in the metal phase to be at 1.1 μm. Both calculated Bragg peak positions agree well with the experimental values, supporting the use of this simple model to describe the reflection behavior of the VO_2 IO.
